# Deep segmentation of 3+1D radar point cloud for real-time roadside traffic user detection

**DOI:** 10.1038/s41598-025-23019-6

**Published:** 2025-11-04

**Authors:** Savankumar Bhanderi, Shiva Agrawal, Gordon Elger

**Affiliations:** 1 Institute of Innovative Mobility (IIMo), Research Group Sensor Technology and Data Fusion for Environmental Perception, Technische Hochschule Ingolstadt, Ingolstadt, 85049 Germany; 2 Applied Center Connected Mobility and Infrastructure, Fraunhofer IVI, Ingolstadt, 85049 Germany

**Keywords:** 3D automotive radar, RoadsideRadar dataset, Deep learning, Intelligent roadside infrastructure, Intelligent transportation system, Radar point cloud, Real-time perception, Smart infrastructure, Engineering, Mathematics and computing

## Abstract

Smart cities rely on intelligent infrastructure to enhance road safety, optimize traffic flow, and enable vehicle-to-infrastructure (V2I) communication. A key component of such infrastructure is an efficient and real-time perception system that accurately detects diverse traffic participants. Among various sensing modalities, automotive radar is one of the best choices due to its robust performance in adverse weather and low-light conditions. However, due to low spatial resolution, traditional clustering-based approaches for radar object detection often struggle with vulnerable road user detection and nearby object separation. Hence, this paper proposes a deep learning-based $3+1$D radar point cloud clustering methodology tailored for smart infrastructure-based perception applications. This approach first performs semantic segmentation of the radar point cloud, followed by instance segmentation to generate well-formed clusters with class labels using a deep neural network. It also detects single-point objects that conventional methods often miss. The described approach is developed and experimented using a smart infrastructure-based sensor setup and it performs segmentation of the point cloud in real-time. Experimental results demonstrate 95.35% F1-macro score for semantic segmentation and 91.03% mean average precision (mAP) at an intersection over union (IoU) threshold of 0.5 for instance segmentation. Further, the complete pipeline operates at 43.61 frames per second with a memory requirement of less than 0.7 MB on the edge device (Nvidia Jetson AGX Orin). We will release the RoadsideRadar dataset along with the code implementation of this work at https://github.com/bhanderisavan/roadside-radar-seg.

## Introduction

Intelligent roadside infrastructure-based perception is an emerging research direction within the field of intelligent transportation systems (ITS) due to its potential to realize safe and robust connected mobility through cooperative perception^1,2^. These systems utilize sensors mounted on roadside infrastructure for achieving efficient, reliable, and real-time environmental perception^3,4^. The most prominently used sensor in these systems is the low-cost RGB camera due to its real-time high-performance object detection and segmentation ability^5–7^. However, their sensing ability degrades considerably during adverse weather and poor lighting conditions. Lidar sensors can overcome these limitations but are unable to deliver real-time scene understanding while also being one of the costliest^8^. Combining the complementary strengths of different sensor modalities by multi-sensor fusion is another method for realizing robust perception. Early fusion approaches operate by combining the raw data from the sensors to achieve richer scene representations but require precise calibration and synchronization. Late fusion, in contrast, integrates the outputs from independent object detectors. While multi-sensor fusion can provide more robust perception, it also increases the overall cost and complexity of the system^9^. Another rather unexplored solution is the next generation $3+1$D mmWave automotive radar, which provides much denser point clouds compared to the previous $2+1$D versions. The additional 1D in both versions refers to Doppler velocity. The radars are used heavily in the autonomous driving field for improving the advanced driver assistance system (ADAS) functions^10,11^ due to their robustness in adverse weather conditions and long-range sensing ability. Additionally, the sparse nature of the radar point clouds requires significantly less computational resources compared to for example the dense point clouds from the lidar sensor. This aligns perfectly with the real-time requirement of the edge devices on the infrastructure-based perception setups.

The perception ability of vehicle on-board sensors is limited by range and occlusions, which can be enhanced by flexible location and pose of infrastructure sensors to increase the on-road safety of traffic participants^12,13^. The information from the roadside perception sensors can be shared with passing vehicles in real-time using vehicle-to-everything (V2X) communication for cooperative driving automation^14^. Furthermore, the infrastructure sensors contribute to traffic flow optimization by automated traffic light control^15,16^, cooperative maneuver planning^17^, traffic surveillance, traffic monitoring, emergency vehicle prioritization, providing warnings to road users, and many more. Albeit the above-mentioned advantages, the research on perception algorithms for such systems is still limited, and confined on the whole to the monocular camera and lidar sensors^1,2^.

**Fig. 1:**
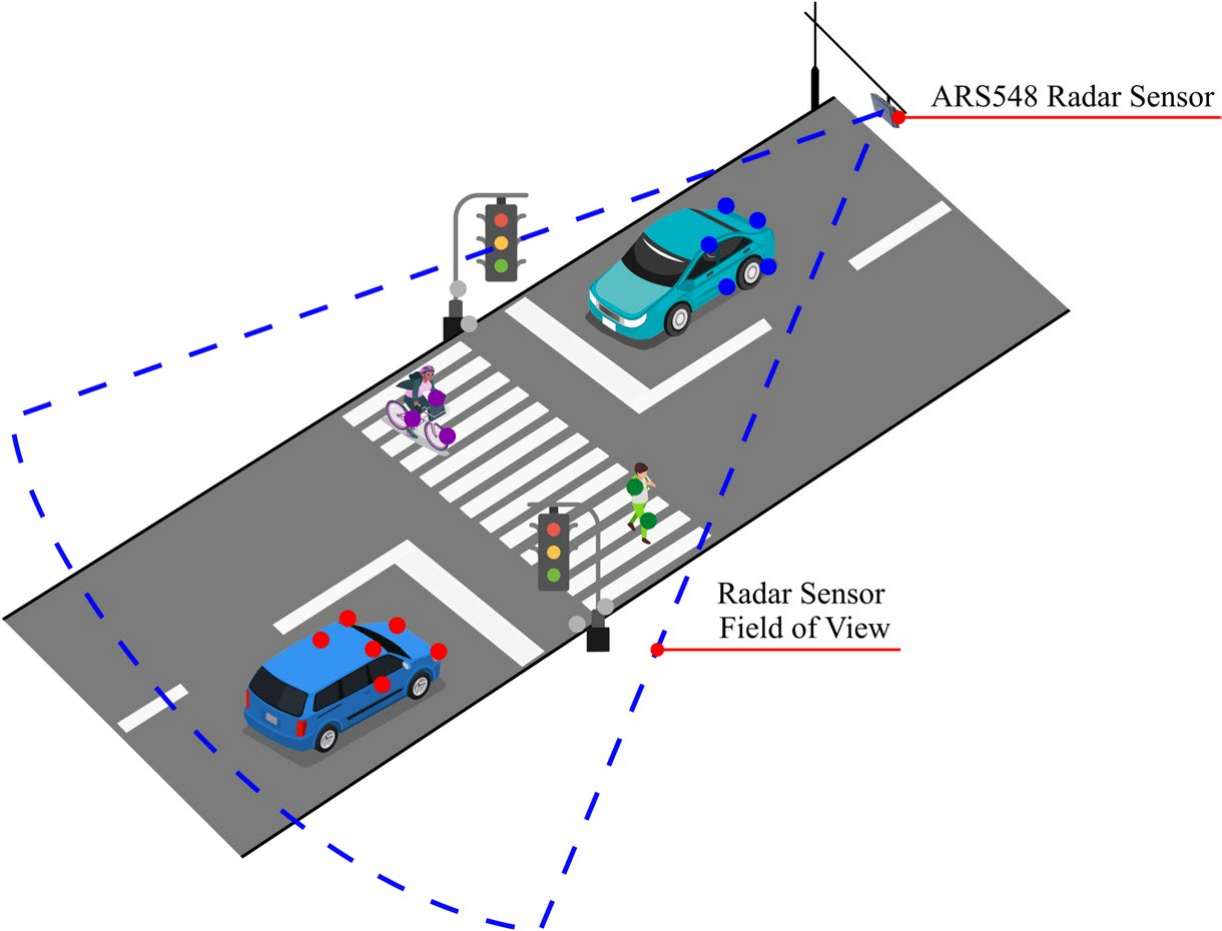
A concept of roadside perception using automotive radars.

In this work, the suitability of automotive radar sensors in the smart roadside infrastructure-based perception task is evaluated through a novel deep learning-based radar point cloud segmentation pipeline. In particular, multi-layer perceptron (MLP) and self-attention mechanisms are used in this work for performing semantic and instance segmentation of key road users such as pedestrians, bicycles, motorcycles, cars, and buses. An example of such a system is presented in Fig. 1, where a $3+1$D radar sensor is mounted on a static infrastructure for detecting various road users.

The key contributions in this paper are listed below.

- A novel deep learning-based 3+1D radar point cloud segmentation architecture for efficient clustering and object detection of road users for static roadside radar sensor.
- Optimization of the segmentation model based on experimental data collected using the Continental ARS548 infrastructure radar sensor. The RoadsideRadar dataset used in this study is made publicly available to encourage further research.
- Implementation and testing of the complete pipeline using the robot operating system (ROS)^18^ on the edge device for real-time performance.

The remainder of this paper is organized as follows. The “Literature Review” Section focuses on current works utilizing machine learning on automotive radar point cloud data. The sensor setup and the dataset used in this work are described in the “Dataset” Section. The “Methodology” Section details the proposed methodology and section “Experiments and Results” provides the implementation details along with experimentation and results. Finally, Section “Conclusion and Future Work” concludes the paper and highlights possible future work.

## Literature review

This section provides a comprehensive summary of the state of research on machine learning applied to automotive radar point clouds. Considering the lack of research utilizing automotive radar sensors in an infrastructure-based setting, the related works on autonomous driving are discussed.

### Semantic segmentation

The traditional methods of scene understanding with automotive radar sensors combined clustering with a subsequent cluster classification step to realize object detection on point cloud data^19–21^. These approaches employ DBSCAN algorithm to cluster raw radar detection points followed by a handcrafted feature generation process for each cluster. Subsequently, these feature vectors are classified using various classifiers such as random forests, long short-term memory (LSTM)^19^, support vector machines (SVM)^20^, and recurrent neural networks (RNN)^21^. These methods deliver suboptimal performance due to their inherent dependency on the accuracy of clustering algorithms and manual feature selection.

Another group of research utilizes pioneering works of PointNet^22^ and PointNet$++$^23^ to realize efficient feature extraction for semantic segmentation. For example, Cennamo et al.^24^ compared PointNet and PointNet++ for moving pedestrian classification using radar point clouds. The same authors provide a neural network architecture for semantic segmentation of $2+1$D radar point clouds in^25^. In another work, Schumann et al.^26^ adapted PointNet$++$ to segment the dynamic $2+1$D radar point clouds in 6 distinct semantic categories.

Following the recent success of self-attention^27^ in the computer vision and natural language processing (NLP) domain, some works have adapted it to work with radar point clouds. For instance, Yu et al.^28^ incorporated self-attention module from point transformer network^29^ with PointNet to perform semantic segmentation on high density simulated 3D radar point clouds. Radar Transformer^30^ is another classification network that is constructed entirely using self-attention modules. Furthermore, Zeller et al.^31^ proposed an encoder-decoder structured network that uses self-attention based up-sampling and down-sampling to accurately perform single scan segmentation of dynamic radar reflections. Similar work is done in^32^ where a velocity transformer layer is introduced to facilitate the separation of static and moving objects.

### Instance segmentation

Schumann et al.^33^ proposed a complete pipeline for scene understanding to detect both static and dynamic objects simultaneously. Static objects are segmented using a convolutional neural network (CNN) on a radar cross section (RCS) histogram grid map from accumulated radar frames. For dynamic objects, an RNN predicts class probabilities and direction vectors, shifting points for better semantic instance alignment. Instance proposals are then generated and classified using PointNet++. Similar approaches with center shift vectors and class-specific clustering are done in^34,35^. In^36^, Yuan^36^ enhanced a U-Net inspired backbone with attention blocks for efficient per-point feature extraction. The instance formation was done using a class-based DBSCAN clustering of the foreground points.

Palffy et al.^37^ enhanced instance segmentation by combining pre-CFAR radar cubes with $3+1$D radar point clouds. Small regions in the radar cube are cropped based on post-CFAR point cloud locations and processed with a 3D CNN for feature extraction to perform semantic segmentation. Again, class-specific DBSCAN is used for instance formation. In^38^, panoptic segmentation is achieved on $3+1$D radar point clouds using PointNet and MLPs. The per-point features are augmented with the azimuth value of each point to facilitate instance id prediction. Zeller et al.^39^ separated static and moving instances by temporally integrating radar point clouds from previous frames with a sequential attentive feature encoding module.

Although significant progress has been made in perception using vehicle-mounted radar sensors, these approaches cannot be directly transferred to roadside radar because of the fundamental differences in the resulting point clouds characteristics. For example, object motion relative to a stationary radar differs significantly from ego-motion–compensated measurements, and occlusion patterns are inverted. A roadside radar often observes traffic participants laterally, rather than from behind. Moreover, vehicle-mounted perception typically prioritizes near-field detection for driver assistance, whereas infrastructure-mounted radars cover larger areas. The fixed nature of roadside radars further enables the use of background subtraction algorithms to segment static road users, which cannot be used with vehicle-mounted sensors. A thorough analysis of the current literature reveals the lack of research on using automotive radar for intelligent roadside perception. In addition, static road users are often overlooked and real-time capabilities of the algorithms are seldom evaluated.

## Dataset

The RoadsideRadar dataset used in this work is an extended version of the INFRA-3DRC dataset^40^. The original INFRA-3DRC dataset is enriched with radar frames in adverse weather and poor lighting conditions.Furthermore, the frames with groups and parking spots are removed from the dataset. The statistical properties of the resulting dataset, along with the roadside sensor setup are elaborated in this section.

### Measurement setup

**Fig. 2:**
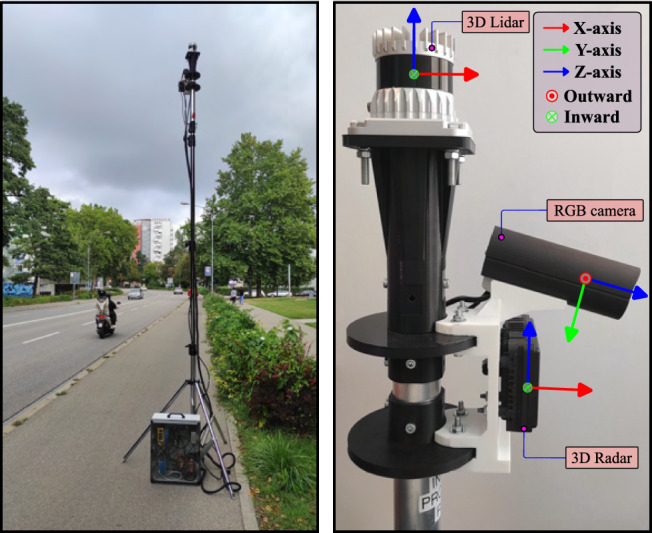
Smart roadside infrastructure-based sensor setup^41^ used in this work.

The data is collected using a **Continental ARS548** $3+1$D radar sensor^42^ mounted at the height of approximately 3.5 meters on the measurement setup shown in Fig. 2. Further details of the measurement setup are provided in^15,16^. Data is collected from two different locations in the city of Ingolstadt, Germany by placing the sensor setup on one side of the road and stored on the disk using the ROS framework.

The locations were chosen to cover two specific geographical conditions suitable for roadside environment perception: 1) a straight road, and 2) a pedestrian crossing intersection. The collected data was post-processed and labeled using the methodology highlighted in^41^. This method requires accurate radar-camera spatial calibration, which was performed following the approach described in^43^. Since the labeling method in^41^ provides annotations only within the radar-camera overlapping field of view, radar points outside this region were discarded. For this work, only the radar data is used for object detection tasks, and images are used for visualization purposes only. It should be noted that the ARS548 radar sensor provides an interface to extract $3+1$D point cloud data only, and the low-level data formats described in^44^ are not accessible. As a result, only the point cloud data is used in this work, which is obtained through the standard signal processing done within the sensor itself. This sensor limitation often results in the loss of information about static road users during the conversion from the low-level signal representation to the sparse point cloud. Consequently, there are frames where the radar sensor fails to deliver points from vulnerable road users (VRUs) even when they are present in the scene. These frames are not considered in the dataset.

### Dataset statistics

Each radar detection point is labeled as one of 6 distinct semantic classes: *background*, *person*, *bicycle*, *motorcycle*, *car* and *bus*.The radar sensor additionally delivers numerous false positives, noise points, and reflections from static surfaces that are irrelevant for object detection. All these detection points that remain after background subtraction are aggregated into a single *background* semantic category. In this work, a road user is annotated as static if it has zero velocity and was not present at the time of background detection, as explained in^41^.

The final dataset comprises 5399 frames of annotated radar point clouds, with over 228*k* labeled radar reflection points and 13292 object instances.

This results in an average of 2.47 annotated objects in each frame. $70\%$ of the frames are used for training, and the remaining $30\%$ are equally divided between validation and testing. Details about the class distribution of the labeled objects in each split are given in Table 1. Further information about the number of static and dynamic points per class is provided in Table 2.

**Table 1:** Summary of the dataset.

	Total points			Total objects		
	Train	Val	Test	Train	Val	Test
Person	4297	896	889	1920	408	392
Bicycle	6517	1403	1274	2723	578	544
Motorcycle	679	140	179	214	47	53
Car	15,964	3337	3612	3786	785	880
Bus	12,483	2420	2632	680	145	137
Background	119,099	26,746	25,507	–	–	–
$\sum$	159,039	34,093	34,942	9323	1963	2006

**Table 2:** Static and dynamic labeled points per class in the dataset.

	Person	Bicycle	Motorcycle	Car	Bus
Static points	285	638	78	2370	0
Dynamic points	5797	8556	920	20,543	17,535
$\sum$	6082	9194	998	22,913	17,535

## Methodology

**Fig. 3:**
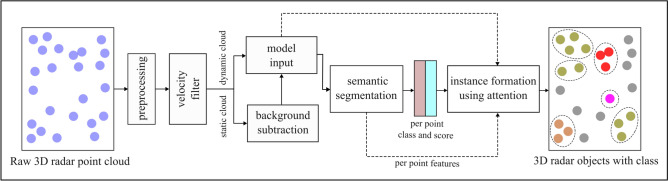
Overview of the proposed pipeline. The semantic and instance segmentation are performed sequentially. Multi-layer perceptrons and self-attention mechanisms are used for semantic and instance segmentation respectively.

Figure 3 highlights the overview of the proposed pipeline for obtaining semantic and instance segmentation on $3+1$D automotive radar point clouds using a roadside sensor setup. Each detection point $D_k$ in the radar point cloud is a row vector such that,

$$\begin{aligned} {D_k} \in \mathbb {R}^{(1\times 5)} (k \in 1,\ldots ,n), \end{aligned}$$

and it can be expressed as:

$$\begin{aligned} {D_k} = [r_k \quad \theta _k \quad \gamma _k \quad v_{d,k} \quad \sigma _k]^T , \end{aligned}$$

where ${r_k}$, ${\theta _k}$, ${\gamma _k}$, ${\sigma _k}$, and $v_{d,k}$ represents range, azimuth angle, elevation angle, RCS, and radial Doppler velocity respectively. Before further processing, the Cartesian coordinates $(x, \; y, \;z)$ are computed from the polar values, and the velocity components $v_x$ and $v_y$ are derived from the Doppler velocity. Additionally, a unique index is assigned to each point to track its order through various processing stages.

To begin with, the raw point cloud from the sensor is passed through a preprocessing step, where the points with implausible Doppler velocity are removed and the field of view is aligned. The minimum and maximum values used for this purpose are shown in Table 3.

**Table 3:** Minimum and maximum values for field of view alignment.

	$\varvec{x \;(m)}$	$\varvec{y \;(m)}$	$\varvec{z \;(m)}$	$\varvec{v_d \;(m/s)}$
Minimum value	0	$-80$	$-4$	$-25$
Maximum value	100	80	1	25

These values were obtained through a thorough analysis of the statistical distribution of each radar feature. The filtered point cloud is then divided into static and dynamic clouds using a Doppler velocity threshold. Static cloud is subjected to 3D radar background subtraction to remove noise and obtain static foreground detection points, as explained in^41^. The resulting foreground points, along with dynamic points form the input to the neural network model, marked with the *model input* block in Fig. 3. The model input is a point cloud containing *N* points, with each point having 6 features, namely *x*, *y*, *z*, $v_x$, $v_y$, and $\sigma$. For efficient and stable training of the neural networks, each input feature is normalized using the *minmax* normalization method^45^. The network first performs semantic segmentation on the model input point cloud, resulting in a class label and score assigned to each point. The per-point class labels, along with the per-point features and model input point cloud are further utilized to obtain instances using the self-attention mechanism.

### Semantic segmentation

The goal of semantic segmentation is to predict a semantic class label for each point in the input point cloud from a set of distinct categories. To achieve this goal, the following challenges must be addressed: (1) The point cloud is unordered set of points, meaning that the algorithm must satisfy the permutation invariance property (i.e. the output of the algorithm must not change with respect to the order of points in the input), (2) The inference time of the algorithm must be low enough to satisfy real-time constraint, and (3) The input point cloud is sparse in nature with limited information, an efficient feature extraction method must be implemented.

**Fig. 4:**
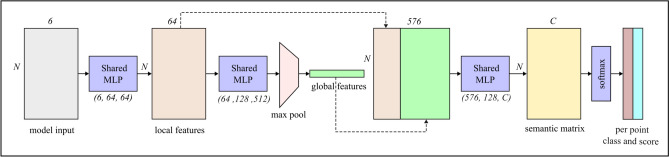
Semantic segmentation network. The architecture used shared MLPs for feature extraction, and is inspired by PointNet^22^.

To tackle the aforementioned challenges, a combination of MLPs and global max pooling is used as depicted in Fig. 4. The MLPs perform local feature extraction using a fully connected layer, and the global max pooling aggregates global context from the entire input point cloud, thereby maintaining the permutation invariance of the point clouds. The structure is inspired by the pioneering work of PointNet^22^, which is generally used for indoor part classification and segmentation. The input to the semantic segmentation block is an array of normalized points, and it outputs per-point class, scores, and per-point features to be used in the next stage. The block diagram of the semantic segmentation network is shown in Fig. 4.

The normalized 6 dimensional input point cloud is first forwarded to a shared MLP which transforms the input to 64 dimensional feature space. Note that the original version of PointNet uses two transformation networks called (T-Net), which predicts an $(N\times N)$ transformation matrix. The output of the first T-Net is used to project input points to its canonical representation before extracting features, and the output of the second T-Net is used for feature alignment in higher dimensional latent space. This is particularly useful when the input is a dense point cloud such as the data in indoor object segmentation and part segmentation, but it does not hold any logical ground for sparse point clouds such as radar data where there is no clear concept of the canonical representation of the entire scene.

Unsurprisingly, the experiments showed that using any of these T-Nets in the algorithm increases the network parameters without affecting the overall performance. Hence, the T-Nets are omitted and the shared MLPs are adjusted for 64 dimensional local features extraction on sparse radar point clouds.

The 64 dimensional per point local features are further mapped to a 512 dimensional feature space using an additional block of MLP. At this stage, the per-point features are represented with a matrix of shape $(N\times 512)$, where *N* denotes the number of points. A column-wise global max pooling operation is applied to this matrix, resulting in a single global feature vector with shape $(1\times 512)$. It is repeated *N* times along the rows to form a global feature matrix of shape $(N\times 512)$ and concatenated with the per point 64 dimensional local features to obtain a combined feature representation of shape $(N\times (64 + 512))$. The global pooling operation serves two purposes: 1) it embeds global context in the learning process, and 2) it ensures that the network is permutation invariant. Subsequently, a segmentation head applies a set of fully connected layers to the combined feature matrix, resulting in an $(N\times C)$ semantic matrix, where *C* denotes the number of classes in the dataset (in this work, it is 6). This matrix represents per-point class-wise logits (pre-activation values), which are passed through a softmax activation layer to convert the logits into probabilities. The final class labels and confidence scores are determined based on the maximum a posteriori probability rule.

These per-point class labels along with class scores and 64 dimensional per point local features are forwarded to the next block of the processing pipeline where the instances are formed using a self-attention-based algorithm.

### Instance segmentation 

**Fig. 5:**
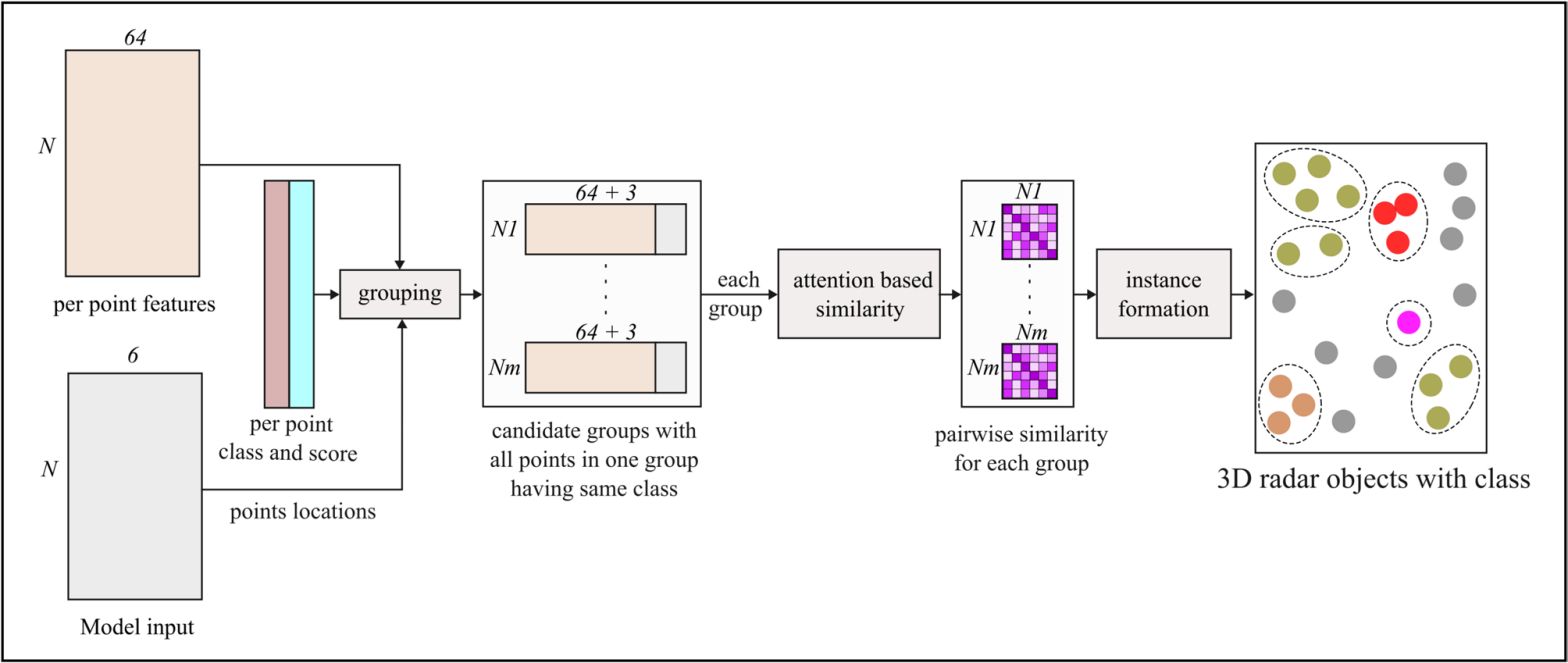
Instance segmentation network. The architecture uses the self-attention mechanism for learning pairwise similarity in the feature space.

After performing semantic segmentation, i.e. a per point class label for each point is obtained, the next step in the processing is to utilize this additional information to generate clusters of points that are reflected from the same object, also known as the instance segmentation. To address the challenge that the number of road users (i.e. instances) is not known beforehand, a similarity learning based instance segmentation algorithm is designed, as presented in Fig. 5. It utilizes a modified version of the self-attention^27^ mechanism for instance separation. The algorithm takes three inputs, namely (1) per point class labels and scores from the previous section, (2) per point 64 dimensional local features from the previous section, and (3) the model input point cloud described in the previous section. It outputs a list of instances (objects) with class labels.

**Fig. 6:**
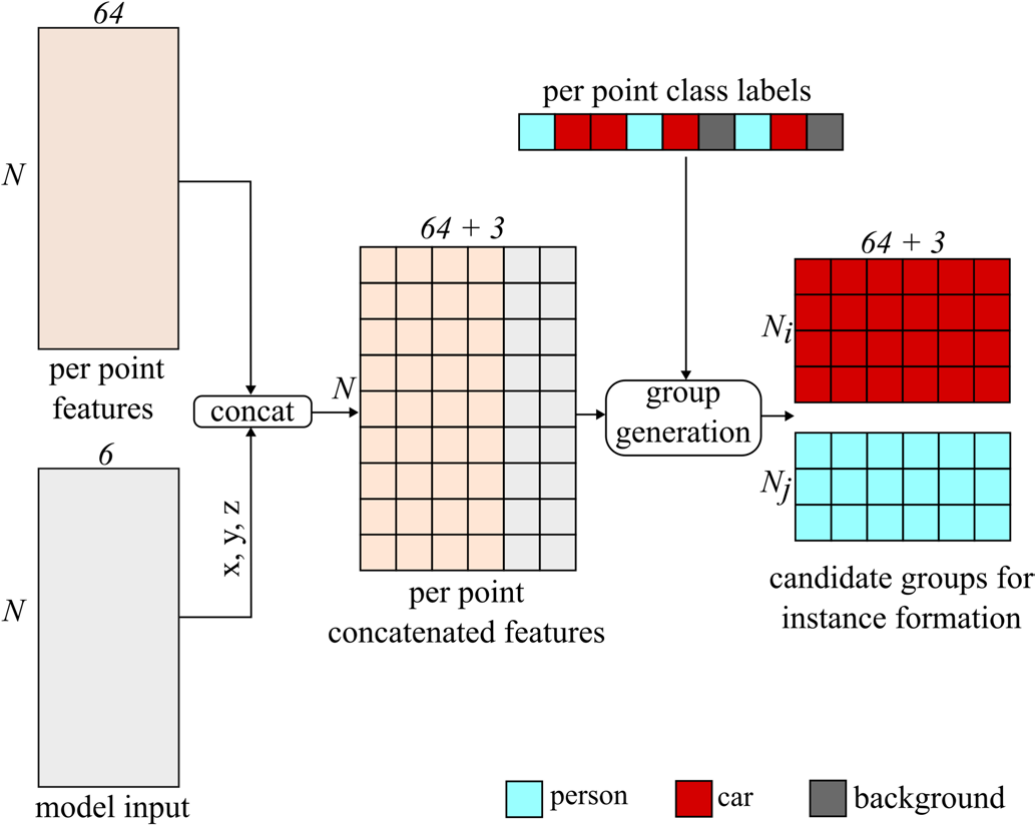
Candidate group generation for instance segmentation.

As shown in Fig. 5, the instance segmentation algorithm is composed of three parts: (1) grouping, (2) pairwise similarity computation, and (3) the instance formation. In the grouping stage, a list of *M* probable candidate groups is generated such that all points in a given group share a common predicted class label. It uses the per-point class labels along with the local features and model input cloud for candidate group generation. The entire process is shown as a block diagram in Fig. 6, and the pseudo-code is provided in Algorithm 1.

**Algorithm 1:**
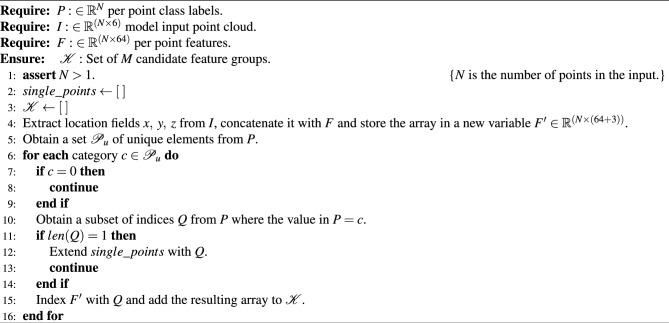
Candidate group generation algorithm.

First, the per-point 64 dimensional local features obtained from the semantic segmentation block are concatenated with the per-point normalized spatial coordinates (*x*,  *y*,  *z*) from the model input cloud. The inclusion of *x*,  *y*, and *z* in the features serve as additional information during the similarity learning process, enabling better instance separation.

Next, the per point class labels are used to split the concatenated features into *M* arrays, where the $i^{th}$ array has a shape of $(N_i \times (64+3))$. Here, $N_i$ is the number of points in the array, 64 is the dimension of local per-point features obtained from semantic segmentation, and 3 represents per-point normalized spatial coordinates. Each of these arrays is regarded as one candidate group. It should be noted that the number of points ($N_i$) varies across the groups. A group is generated for each unique predicted class label except for the background class (class label 0) because the concept of *instances* does not apply to it. Furthermore, the total number of formulated groups (*M*) depends on the number of unique predicted class labels in the semantic segmentation output, meaning that the maximum value it can take is the number of distinct foreground class labels (5) in the dataset. In addition, groups are not generated when there is only one unique prediction point for a given class, but such points are considered as instances with one radar reflection.

The next step is to calculate a pairwise similarity matrix for each of the *M* candidate groups using a modified self-attention mechanism. The self-attention^27^ is a common technique in many neural networks to capture the inter-dependencies between different elements of the input sample. It first projects the input to three distinct matrices, famously known as the Keys (*K*), Queries (*Q*), and Values (*V*), using three linear layers. Then the attention weights (scores) are computed using a scaled dot product between Queries (*Q*) and Keys (*K*), and the resulting matrix is passed to softmax activation for row-wise normalization. The scaling factor $\sqrt{d_k}$ is used in the self-attention mechanism to prevent the attention scores from becoming too large, leading to stability during training. These weights are used to take a weighted sum of the Values (*V*), leading to a new set of feature vectors where each point’s representation is enhanced by the information from other points it attends to. The mathematical formulation of self-attention is given in Section 3.2.1 of^27^.

In this work, the self-attention mechanism is modified to model the similarity between a pair of radar points, given that both points belong to the same class. The self-attention weights resulting from the scaled dot product of Queries (*Q*) and Keys (*K*) can be viewed as pairwise similarity scores representing the degree of similarity between a pair of elements. If the attention score between the elements *(i, j)* is high, it suggests that there exists a stronger relationship between these points. It is important to note that this work focuses on evaluating the relationships between pairs of points, and not on aggregating or combining their feature information. This task does not require generating new feature representations for each point based on its relationship with other points, which is the purpose of using the Values matrix. Therefore, the attention weights are considered as an end goal for measuring the similarity between points, and the Values (*V*) are not computed in this work. This decision simplifies the model and reduces the computational complexity.

Furthermore, the standard self-attention uses softmax activation on the attention weights to calculate the importance of each input element on other elements to ensure that the attention weights for each point (row) sum to 1. This means that the sum contribution of all points in calculating an enhanced representation of a given point should not exceed 1. This is useful in contexts where the model needs to make a relative comparison among all points, such as in NLP where the relationship between different words in a sentence is modeled. However, it is not useful in this work because here the task is determining the similarity between pairs of points, specifically evaluating whether they belong to the same road user. In this case, an independent, binary-like decision about the similarity between pairs of points is needed. Therefore, the softmax activation is replaced by the sigmoid activation, which provides a straightforward way to determine whether two points are likely from the same road user without needing to normalize across other points.

**Algorithm 2:**
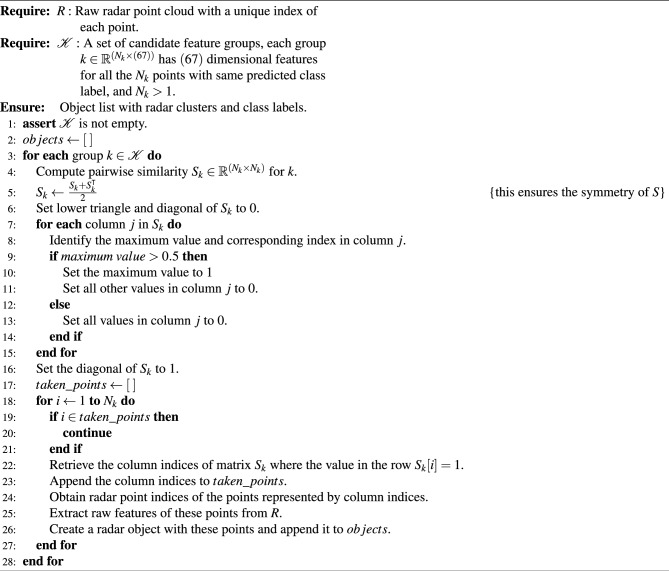
Instance formation algorithm.

**Fig. 7:**
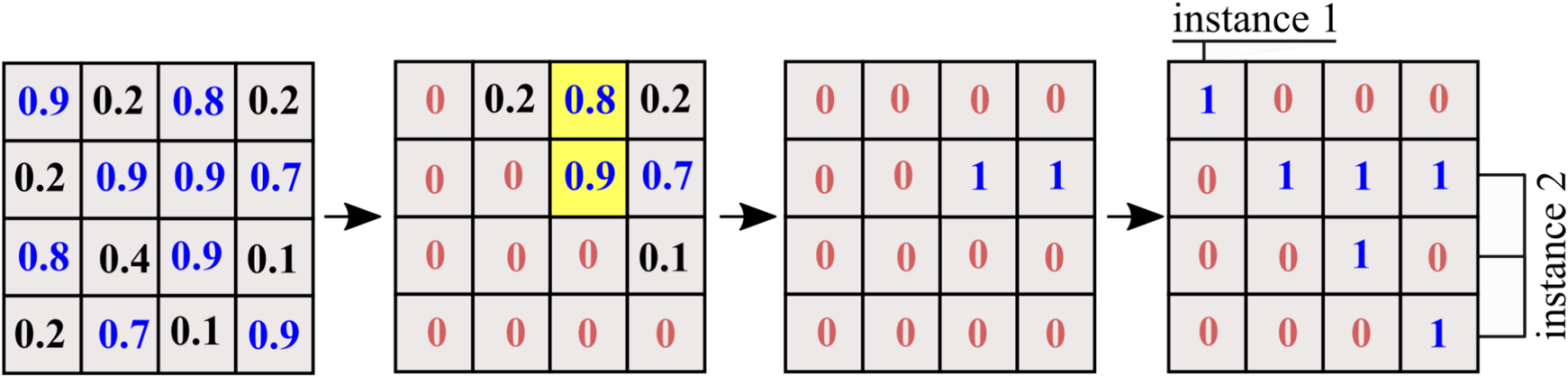
Instance formation from predicted similarity map. Left to right: predicted pairwise similarity map $S_{sym} \in \mathbb {R}^{(N_i \times N_i)}$ (made symmetric by averaging across the diagonal), bottom triangle zeroing, per-column max thresholding, instance generation.

Considering the above-mentioned modifications, one self-attention weight map (also referred to as a pairwise similarity map) is calculated for each of the *M* candidate groups. For a given group with $N_{i}$ radar points, the similarity map $S_{sym} \in \mathbb {R}^{({N_i}\times {N_i})}$ is made symmetric by averaging across the diagonal before further processing. Furthermore, the lower triangle and the diagonal of the matrix $S_{sym}$ is zeroed for faster computation. In the next step, the $S_{sym}$ matrix is converted into a binary matrix for instance extraction. First, the maximum values in each column are replaced with 1 if it is greater than 0.5 and all other values in the same column are replaced with zero. The resulting binary similarity map has either zero or exactly one element in each column with the value of 1. This resolves an ambiguity in instance assignment where one or more points are probable candidates for two or more instances. At this stage, the diagonal elements of the similarity matrix is set to 1 to simplify further processing. This process is illustrated in Fig. 7. The ambiguity case is highlighted with yellow color.

For each row (point) in the binary similarity matrix, the index locations of the columns which has a similarity of 1 with the current row (point) can be obtained. This can be interpreted as the current point and the points with a similarity of 1 with the current point belong to the same instance. Instances can be formed using these index locations and the raw radar point cloud. The processing for the current row is skipped if its index is already assigned to an instance. These indices are used to query the model input point cloud and construct actual instances of the 3D radar point cloud with class labels. The predicted score of an instance is calculated as the mean of the scores of all points that belong to the instance. The pseudo-code for the instance extraction process using candidate groups is shown in Algorithm 2.

## Experiments and results

This section provides the implementation details of the proposed method along with the quantitative and qualitative results of the best-performing model on a test set. Ablation studies and experiments are also provided.

### Implementation

The training was conducted in an end-to-end manner on a machine equipped with a single NVIDIA RTX 4090 GPU using PyTorch^46^ library. The network comprises 164*K* trainable parameters, with a memory size of 0.628 MB. The network was trained for 100 epochs. One epoch takes approximately 90 s to process, including data loading, training, and validation. The dataset imbalance was addressed by using a weighted categorical cross-entropy loss for the semantic segmentation task. For instance segmentation task, binary cross entropy loss (BCE) was calculated for each candidate group and averaged to obtain a scalar value. The mathamatical formulation of the loss functions are given below.

1 $$\begin{aligned} {\mathscr {L}_{total}} = \lambda _{sem\_seg} \cdot \mathscr {L}_{sem\_seg} + \lambda _{inst\_seg} \cdot \mathscr {L}_{inst\_seg} \end{aligned}$$

where ${\mathscr {L}_{total}}$ is the total scalar loss value for a single radar frame, $\lambda _{sem\_seg}$ is the weight assigned to semantic segmentation task (1 is used in this work), $\lambda _{inst\_seg}$ is the weight assigned to similarity learning (instance segmentation) task (2 is used in this work), ${\mathscr {L}_{sem\_seg}}$ is the classification loss for a single radar frame, and ${\mathscr {L}_{inst\_seg}}$ is the similarity loss for a single radar frame.

2 $$\begin{aligned} \mathscr {L}_{sem\_seg} = -\frac{1}{N} \sum _{n\in N}\sum _{c\in C}{w_c} \cdot {y_{nc}} \cdot \log ({\hat{y}_{nc}}), \qquad {w_{c}} = \frac{M}{C \cdot {M_c}} \end{aligned}$$

where *N* is the number of points in a radar frame, *C* is the total number of classes in the dataset, $w_c$ is the loss weight for the class *c*, ${\hat{y}_{nc}}$ is the predicted probability of point *n* belonging to class *c*, $y_{nc}$ is a binary indicator that is 1 if class label *c* is the ground truth class label for point *n*, *M* is the total number of points in the entire training dataset, *C* is the number of classes, $M_c$ is the number of points belonging to class *c* in the dataset, and $w_c$ is the class-specific loss weight for class *c*.

3 $$\begin{aligned} \mathscr {L}_{inst\_seg} = \frac{1}{M} \sum _{i\in G} \mathscr {L}_{BCE_i}, \qquad \mathscr {L}_{BCE} = -\frac{1}{N} \sum _{n\in N} [{y_{n}} \cdot \log (\hat{y}_{n}) + (1 - {y_n}) \cdot \log (1 - \hat{y}_{n})] \end{aligned}$$

where *M* is the total number of instance formation candidate groups, $\mathscr {L}_{BCE_i}$ is the binary cross entropy loss for the group *i*, *N* is the total number of points in an instance formation group, ${y_n}$ is the ground truth class label for the point *n*, and $\hat{y_n}$ is the predicted probability of point *n* belonging to the positive class.

The radar point cloud contains a varying number of points across different timestamps, which poses a challenge in training the neural network models using mini-batch gradient descent. This challenge was addressed by using the packing and padding functionality provided by the PyTorch^46^ library. During the max pooling operation, the sequence is padded with the value of negative infinity, which effectively ignores the padded points while calculating the max value for each feature.

**Fig. 8:**
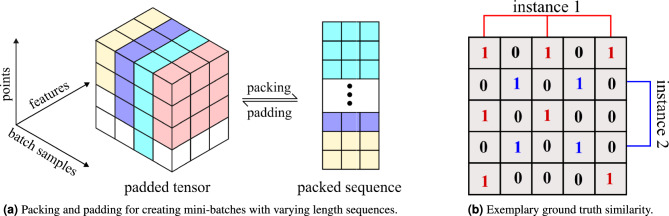
Data preparation for training.

The process of packing and padding is illustrated in Fig. 8a. The ground truth similarity maps are generated dynamically based on the output of the semantic segmentation branch. An example ground truth similarity map with two ground truth instances is shown in Fig. 8b. The first instance (shown in red) consists of points with indices (1,3,5) and the second instance(shown in blue) consists of points with indices (2,4). The most important training parameters are given in Table 4.

**Table 4:** Training parameters.

Parameter	Value
Batch size	64
Optimizer	Adam
Learning rate	0.0003
Weight decay	0.0002
Gradient clipping	3
Normalization	*LayerNorm*
Activation	*LeakyReLU*
LR scheduler	*ReduceLROnPlateau*

### Performance evaluation

**Fig. 9:**
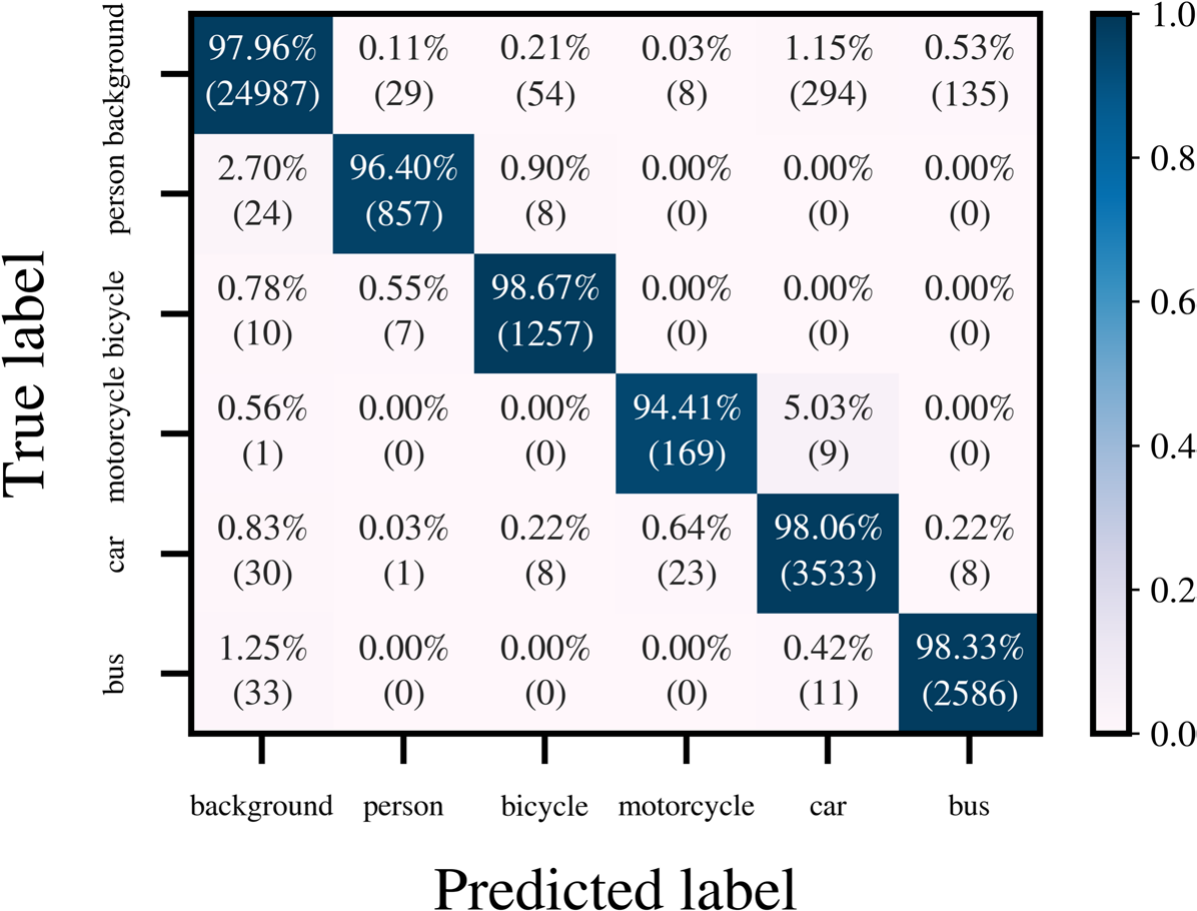
Confusion matrix obtained through the test set.

The performance of the proposed method was evaluated on a dedicated test set. For semantic segmentation, the confusion matrix for best-performing architecture on the test set is presented in Fig. 9, where the majority of correct predictions are concentrated along the diagonal, indicating strong overall performance in detecting true positive radar points. Particularly, the *background* has the highest correct classifications (24, 987), though there are some incorrect predictions, with 294 *background* instances predicted as *car* and 135 as *bus*. Additionally, 24 *person*, 30 *car*, and 33 *bus* points were incorrectly classified as *background*. Such errors in predictions could be attributed to the similarities in radar signatures between static objects and other classes. Furthermore, static objects with low velocities can easily be confused with *background*. The class-wise and macro averaged $F_1$ scores are shown in Table 5. Overall, the network achieves a $F_1$ score of $95.35\%$, indicating the model’s effective performance across diverse classes.

**Table 5:** Performance evaluation results. Semantic segmentation task (per point level) is evaluated using the $F_1$ score. Instance segmentation task is evaluated using AP at varying IoU thresholds. The value of each metric is reported in %. Bold shows the overall performance averaged across classes.

	$\varvec{F}_1$	AP^.30^	AP^.50^	AP^.75^	AP
Background	98.78	–	–	–	–
Person	96.13	94.80	92.67	84.10	84.83
Bicycle	96.65	94.28	92.44	84.73	85.16
Motorcycle	89.18	95.73	91.03	85.27	82.83
Car	94.85	94.33	93.00	77.73	76.90
Bus	96.51	87.63	86.03	75.51	70.35
**Average**	**95.35**	**93.36**	**91.03**	**81.47**	**80.01**

For instance level evaluation, a commonly used metric is the mean average precision (mAP), which utilizes a specific intersection over union (IoU) threshold for assigning ground truth to predictions. For 3D point-based algorithms, IoU is calculated on point sets instead of areas or volumes as described in^47^. For radar point clouds, 0.3 and 0.5 are the most prominently used IoU thresholds^47^. In addition to these thresholds, the proposed network is evaluated at an IoU of 0.75. Furthermore, the COCO AP formula^48^ is used for calculating the overall mAP, which considers the individual class average precision (AP) at a range of IoU thresholds between 0.5 and 0.95 for ensuring optimization across different levels of detection difficulty. The resulting statistics for each class along with the macro average are provided in Table 5. In the table, the $F_1$ score is calculated at a per-point level as explained previously and the remaining metrics are calculated at an instance level. Furthermore, the $AP^i$ refers to average precision at an IoU threshold of *i*, and *AP* without a superscript follows the COCO AP formula. The mAP for each column except the $F_1$ score is highlighted in the *average* row.

At the lowest IoU threshold of 0.30, the model achieves a high mAP of $93.36\%$, indicating the effective instance separation with moderate overlap between predicted and ground truth objects. This performance is maintained at the IoU $=0.5$, though there is a slight decrease of $\approx {2\%}$ in the AP, and reduces noticeably at the IoU threshold of 0.75 with an AP of $81.47\%$. This highlights the increased challenges of achieving higher overlap for instance segmentation. The COCO mAP shows consistent performance across all classes, suggesting the model maintains reasonable accuracy across various levels of overlap without overfitting to a specific IoU threshold. A closer analysis of the class-specific AP values reveals that the drop in mAP with increasing IoU can be mainly attributed to the *bus* class AP, which consistently has the lowest AP among all classes for all IoU thresholds. This is likely due to a vast difference in the length of the busses in the dataset.

**Fig. 10:**
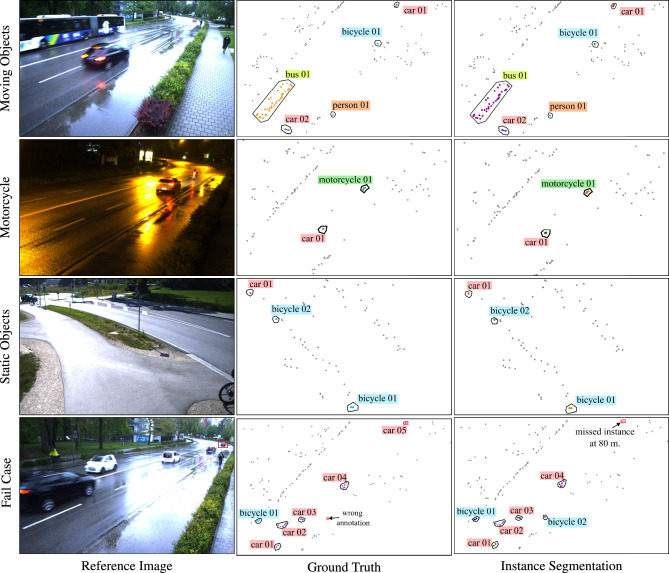
Network predictions visualized in the bird’s-eye view where the elevation values are considered as zero. The reference images are shown for visualization only. The background points are marked with smaller size and grey color for better visualization. See text for details regarding point colors.

**Fig. 11:**
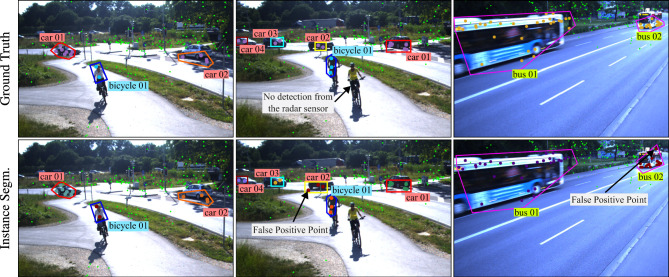
Network predictions projected on image plane where the differences in elevation is clearly visible. The images are shown for visualization purpose only. The background points are marked with smaller size and green color for better visualization. See text for details regarding point colors.

The qualitative results of the proposed approach are highlighted in Figs. 10 and 11. In both figures, individual detection points in the ground truth and semantic segmentation are colored with class-specific colors: goldenrod for bus, purple for car, salmon for person, deepskyblue for bicycle, and cadetblue for motorcycle class. The ground truth class labels are marked using actual annotation files, and the instance segmentation class labels are obtained using the network output. Furthermore, reflections from individual objects in the instance segmentation prediction are marked with a unique color for better interpretation of multiple road users of the same class. Please note that images are shown for visualization purposes only.

In Fig. 10, the network predictions are shown in the bird’s-eye view, where the network efficiently predicts moving as well as static road user instances. This performance is maintained even in the case of the underrepresented motorcycle class and poor lighting conditions, as seen in the second row of the figure. In the last row, the network fails to detect a car at a distance of 80 meters, marked with a red bounding box in the image. A closer inspection of the last sample reveals a false positive *bicycle* (*bicycle 02*) instance. It is labeled as background in ground truth even though it is clearly visible in the reference image. Further investigation in the raw point cloud indicated the errors in ground truth annotations (marked with a red circle in ground truth).

The results shown in Fig. 10 are analysed in the bird’s-eye view, where the elevation value of each point is considered as zero. To better understand the network predictions, Fig. 11 displays some examples of the network’s outputs projected onto the image plane, where the elevation of radar points is better visualized. Each column represents one sample. In the second and third columns, there is one false positive point from *car 02* and *bus 02* respectively. Additionally, the second column demonstrates an inherent limitation of the radar sensor where there is no reflection point from one of the bicycles.

### Comparison with different methods

The comparison of various methods is provided in Table 6. The PointNet^22^ model was modified to perform semantic segmentation on $3+1$D radar point cloud data. Specifically, the input dimension of the PointNet segmentation network was adjusted from 3 to 6 to accommodate the additional features provided by the radar sensor. Additionally, the number of points in each radar frame was fixed to train the model with higher batch sizes. All other parameter values were kept the same as in the default architecture of the PointNet segmentation network provided in^22^.

**Table 6:** Comparison of various approaches. The values of each metric is reported in %. Bold highlight the best performing model for each evaluation metric.

Method	F_1_	mAP^.50^	mAP^.75^	mAP
PointNet^22^	90.12	−	−	−
DBSCAN^49^ + PointNet^22^	−	72.60	67.70	66.90
PointNet^22^ + DBSCAN^49^	90.12	81.96	76.70	74.80
PointNet^22^ + Instance formation	90.12	84.60	79.80	78.68
**This work**	**95.35**	**91.03**	**81.46**	**80.01**

In another implementation, DBSCAN^49^ clustering and PointNet classification network^22^ were used sequentially for obtaining instance segmentation results. Note that this implementation does not provide semantic segmentation of the input point cloud. For clustering, five-dimensional input features were considered, which included 3 spatial dimensions along with 2 Cartesian velocity components. The DBSCAN was configured with $min\_points$ set to 2, and $search\_radius$ set to a uniform value of 3 in each dimension. The resulting clusters were classified using the classification variant of the PointNet model.

Two additional combinations were explored that provide both semantic as well as instance segmentation results. In both methods, the aforementioned modified segmentation network of PointNet was used for obtaining semantic segmentation of the radar point clouds, leading to the identical $F_1$ score as shown in Table 6. For instance segmentation, the first method employed a class-specific DBSCAN clustering algorithm while the second approach used the instance formation algorithm proposed in this paper. Unsurprisingly, the latter approach outperformed the former by $\approx 4\%$ in terms of mAP. The proposed method performed the best with 80.01% mAP.

### Ablation studies

Table 7 highlights the impact of different input radar features on the neural network’s performance. As expected, the combination (*x*, *y*) yields the lowest mAP of $40.60\%$, which is increased by $\approx 23.5\%$ when the elevation (*z*) dimension is added to 2D information. Further enhancement is observed when extending the 2D spatial coordinates with Doppler velocity ($v_d$) and rcs, which boosts the mAP to $66.99\%$. Transforming the radial velocity to Cartesian coordinates ($v_x,\;v_y$) provides an additional improvement, increasing the mAP to $71.42\%$. Additional gains are seen with the integration of radial velocity and elevation information, leading to an mAP of $73.72\%$ with the feature set $(x,\;y,\;z,\;v_d)$. Again, dissolving $v_d$ to $v_x$ and $v_y$ further improves the mAP by $\approx 6\%$.

**Table 7:** Evaluation results on various combinations of the input features. The value of each metric is reported in %. Bold highlights the best performing combination of the input features.

Input fields	F_1_	mAP^.50^	mAP^.75^	mAP
${[x,\;y]}$	77.20	53.85	40.00	40.60
${[x,\;y,\;v_d,\;rcs]}$	92.56	84.26	67.88	66.99
${[x,\;y,\; v_x,\;v_y,\;rcs]}$	93.25	87.86	72.96	71.42
${[x,\;y, \;z]}$	89.44	76.01	64.15	64.10
${[x,\;y,\;z,\;v_d]}$	94.73	87.11	72.99	73.72
${[x,\;y,\;z,\; v_d,\;rcs]}$	95.14	88.31	78.62	77.16
${[x,\;y,\;z,\;v_x\;v_y]}$	95.34	89.41	81.21	79.35
${[x,\;y,\;z,\; v_x,\;v_y,\;rcs]}$	**95.35**	**91.03**	**81.46**	**80.01**

Incorporating rcs in the feature set $(x,\;y,\;z,\;v_d)$ leads to a $4\%$ increment in the AP. Finally, the highest mAP of $80.01\%$ is achieved with the feature set $(x,\;y,\;z,\;v_x,\;v_y,\;rcs)$, surpassing all considered combinations of the input features.

### Runtime evaluation

For runtime evaluation, the entire pipeline shown in Fig. 3 was implemented in ROS^18^ for real-time inference on an edge device (Nvidia Jetson AGX Orin^50^). The inference was conducted with the batch size of 1. The time taken for the entire process, starting from raw data acquisition to the radar object list creation was calculated for numerous scenes with varying numbers of road users and averaged to asses the real-time capability of the system. Furthermore, the time taken by each component was also measured in a similar fashion. The resulting values are shown in Table 8, where the CPU and GPU runtimes of two devices are compared for the proposed method.

**Table 8:** Inference timing of various components in the proposed perception pipeline. The values in all columns except *Hz* are reported in milliseconds.

Hardware	Data Proc.	Sem. Seg.	Inst. Seg.	${\sum }$	Hz
GPU					
Nvidia AGX Orin	5.60	3.28	14.05	22.93	43.61
Nvidia RTX 4090	1.84	1.08	2.24	5.16	193.80
CPU					
Nvidia AGX Orin	4.50	24.56	9.67	38.73	25.82
AMD Ryzen 5975WX	2.20	1.21	1.35	4.76	210.08

The overall runtime of the entire pipeline is 22.93 milliseconds on an edge device with GPU, and the deep learning model takes only 17.33 milliseconds. Implementing the pipeline on the same device with CPU suggests a significant increase in semantic segmentation time. In contrast, the instance segmentation time reduces when using CPU instead of GPU for both devices. This is due to the fact that the semantic segmentation mainly consists of MLP operations, which are implemented using PyTorch *nn.Module* class. The parallel processing of the GPU is much faster in carrying out these operations compared to the CPU. On the other hand, the only neural network operation involved in instance segmentation is the similarity computation using attention, the rest of the process comprises general-purpose computations, including frequent data manipulation and control sequences like for loops. These operations cannot be easily parallelized, leading to longer inference time on GPU compared to CPU.

## Conclusion and future work

In this work, a robust deep learning-based object detection pipeline using point cloud segmentation for the roadside $3+1$D automotive radar sensor is proposed. The network has been trained and evaluated on an extended version of the INFRA-3DRC dataset. The proposed method achieves high values of $91.03\%$ for object level $\mathrm {mAP_{IoU=0.5}}$ and $95.35\%$ for point level $F_1$ score, demonstrating the effectiveness of deep learning applied to roadside radar perception. In addition, the network performance remained robust across various IoU thresholds, as verified by $80.01\%$ COCO mAP. The experiments showed that the additional height information provided by the radar sensor significantly boosts the performance. Moreover, the average inference time of the entire perception pipeline, including the deep learning model, on an edge device (Nvidia Jetson AGX Orin) is 22.93 ms, resulting in a high frame rate of 43.61 frames per second. This highlights that the proposed method can perform real-time scene understanding on an edge device with limited computation.

The future work includes implementing object tracking using statistical filters to smooth the fluctuation in per-frame detection results and enlarging the dataset by covering more geographical locations with a variety of road users.

## Data Availability

The dataset generated and/or analysed during the current study are available at https://github.com/bhanderisavan/roadside-radar-seg.
